# Sequential radiofrequency–pulsed field ablation of a refractory right posteroseptal accessory pathway using a dual-energy catheter

**DOI:** 10.1007/s10840-026-02374-y

**Published:** 2026-06-15

**Authors:** Maicol Cortez-Sandoval, Rodolfo San Antonio, Jesús Rodríguez-Silva, Alex Carrasco, Julián Rodríguez-García, Andrea Di Marco, Paolo D. Dallaglio, Ignasi Anguera

**Affiliations:** 1 Cardiology Department, Bellvitge University Hospital, L’Hospitalet de Llobregat, Feixa Llarga st., 08907 Barcelona, Spain; 2 Cardiovascular, Respiratory and Systemic Disease and Cellular Aging Program, Institut d’Investigació Biomèdica de Bellvitge (IDIBELL), BIOHEART Group, L’Hospitalet de Llobregat, Barcelona, Spain; 3 Abbott, Barcelona, Spain

A 26-year-old man with manifest Wolff-Parkinson-White syndrome and mildly reduced left ventricular ejection fraction (50%) was referred for catheter ablation. The delta-wave morphology suggested a right posteroseptal accessory pathway. The electrophysiological study confirmed a negative HV interval (− 20 ms) and bidirectional pathway conduction, with anterograde and retrograde effective refractory periods of 290 and 280 ms, respectively.

Electroanatomic mapping in sinus rhythm localized the earliest ventricular activation to the right posteroseptal region, approximately 9 mm from the His bundle. The decisive finding was not timing alone, but the local electrogram architecture: atrial signal, short isoelectric segment, discrete accessory pathway potential, and ventricular signal 20 ms before delta-wave onset (Panel A). This A-isoelectric-AP-V sequence is characteristic of recording at the ventricular exit of an obliquely oriented pathway, in which the isoelectric segment reflects conduction from the atrial insertion to a deeper ventricular breakthrough. The pathway was therefore suspected to follow a deep, intramural, or near-epicardial course, despite an endocardially accessible exit [1].

Ablation was performed with an irrigated dual-energy catheter (TactiFlex Duo Ablation Catheter, Abbott), capable of delivering both radiofrequency (RF) and pulsed field ablation (PFA). RF was applied first at 40 W (maximum 43 °C, up to 60 s). The first application abolished pre-excitation after approximately 10 s, but conduction recurred shortly after energy cessation. Repeated RF applications at the target and at adjacent sites reproduced this pattern: prompt but non-durable block (Panel B). This reproducible response argued against a localization error and instead pointed to a depth problem: the catheter had engaged the correct ventricular exit, but RF could not create a lesion deep enough to reach the obliquely oriented fibers.

With the target electrophysiologically validated, the strategy was changed to PFA without remapping. Low-energy PFA was delivered through the same catheter at the same site (Panel C). Pre-excitation disappeared immediately with the first application and did not recur during the post-ablation waiting period. Post-ablation voltage mapping showed a small, contained low-voltage area centered on the target and separated from the His region (Panel D). No atrioventricular conduction disturbance occurred, and follow-up showed no recurrence of manifest pre-excitation.

This case highlights a clinically useful distinction in posteroseptal accessory pathway ablation: failure of durability is not always failure of localization. Posteroseptal pathways are especially prone to this scenario because the apparent endocardial breakthrough may be spatially separated from the pathway’s atrial insertion or intramural body by the three-dimensional anatomy of the region—the inferior pyramidal space, coronary sinus musculature, the middle cardiac vein, epicardial fat, and the converging atrioventricular annuli [1]. In this setting, transient RF block becomes a real-time physiological test: immediate abolition confirms proximity to the relevant tissue, whereas early recurrence signals insufficient lesion depth rather than mislocalization. Sequential, colocalized RF and PFA experimentally create deeper, larger lesions than either alone, with prior RF possibly lowering the electroporation threshold by altering tissue impedance—a rationale for PFA rescue at the RF-validated site [2]. Importantly, emerging clinical data suggest that PFA can be delivered near the atrioventricular conduction system without producing atrioventricular block when appropriate energy settings are used [3], a critical safety consideration in the posteroseptal region.

In a right posteroseptal accessory pathway with electrogram features suggesting a probable deep oblique course, transient suppression during RF delivery identified the correct ventricular exit but also revealed inadequate lesion depth. Sequential low-energy PFA at the same validated site achieved immediate and durable pathway elimination. This case supports a mechanism-guided dual-energy strategy, in which the electrophysiological response to RF helps identify patients in whom transition to PFA is reasonable (Fig. 1).

**Fig. 1:**
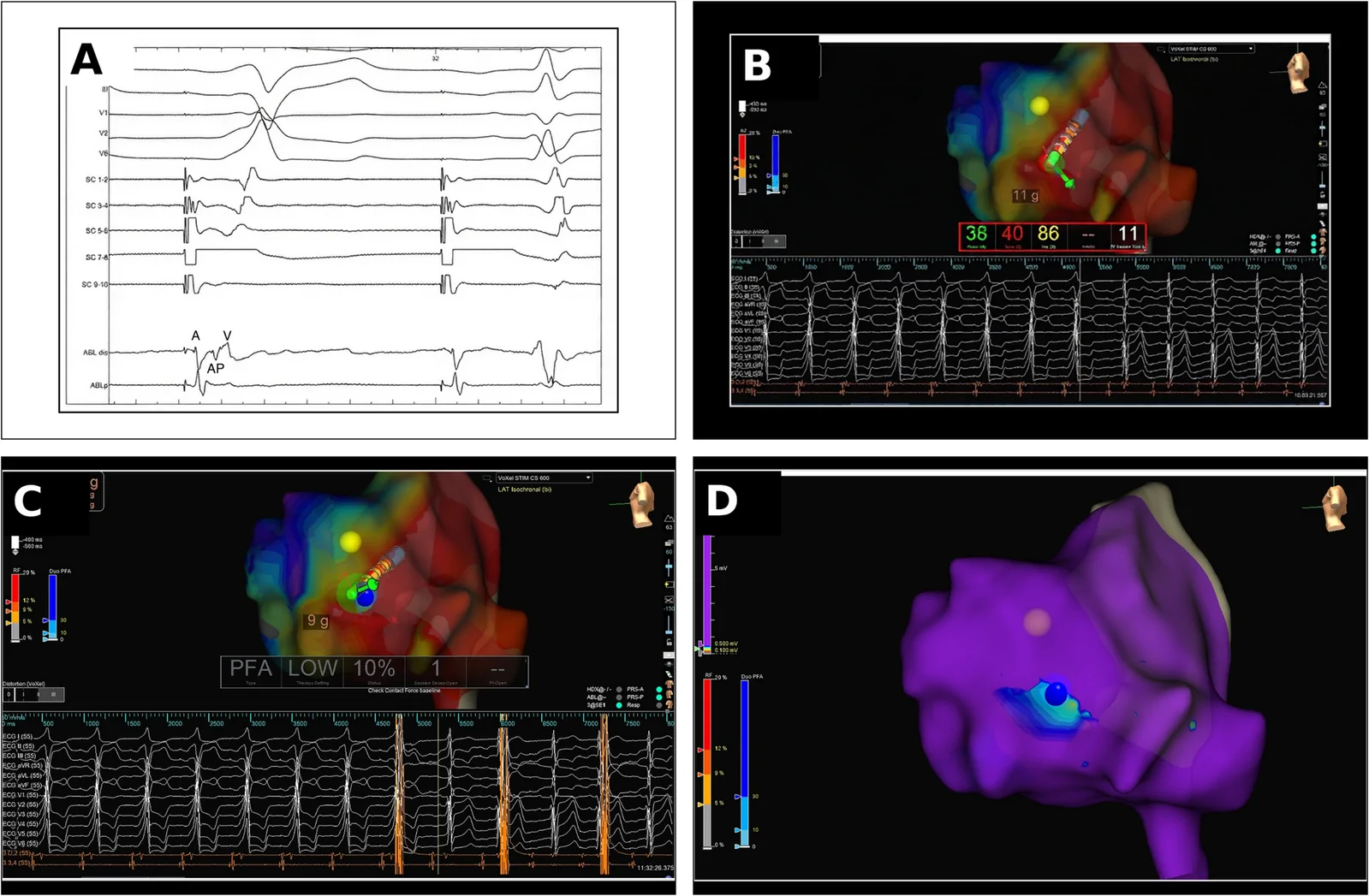
Sequential RF-PFA ablation of a refractory right posteroseptal accessory pathway. (**A**) Target electrogram showing atrial signal, isoelectric interval, accessory pathway potential, and earliest ventricular activation 20 ms before delta-wave onset. (**B**) RF applications produced reproducible but transient pre-excitation suppression. (**C**) Low-energy PFA delivered at the same site using the dual-energy catheter produced immediate pathway abolition. (**D**) Post-ablation voltage map showing a localized low-voltage lesion at the target, separate from the His region. Yellow sphere, His bundle

## Data Availability

No datasets were generated or analysed during the current study.
